# Artificial intelligence–enabled liquid biopsy in cancer: a systematic review and meta- analysis of diagnostic performance and biological implications

**DOI:** 10.3389/fonc.2026.1850705

**Published:** 2026-06-17

**Authors:** Luisana Sisca, Mariam Grazia Polito, Emy Sisca, Michele Iuliani, Alessio Cortellini, Bruno Vincenzi, Giuseppe Tonini, Francesco Pantano

**Affiliations:** 1 Medical Oncology Department, Fondazione Policlinico Universitario Campus Bio-Medico, Rome, Italy; 2 Translational Research Department, Institut Curie, Paris, France; 3 UOC Oncologia Territoriale, ASL Latina – CDS Aprilia, Polo Pontino, Sapienza University of Rome, Latina, Italy; 4 Department of Management Engineering, University of Calabria, Rende, Italy; 5 Department of Surgery and Cancer, Imperial College London, London, United Kingdom

**Keywords:** artificial intelligence, biomarkers, cancer diagnosis, circulating tumor DNA, liquid biopsy, machine learning, meta-analysis, precision oncology

## Abstract

**Background:**

Liquid biopsy offers a minimally invasive approach for cancer detection and monitoring, but its diagnostic performance is often limited by low signal abundance and biological noise. Artificial intelligence (AI) has been proposed as a computational framework to integrate heterogeneous and biologically noisy circulating biomarker data; however, the biological underpinnings, clinical relevance, and translational meaning of the reported performance gains remain unclear.

**Methods:**

We conducted a systematic review and random-effects meta-analysis of studies published between 2022 and 2025 applying AI-based methods to liquid-biopsy data for oncologic diagnosis. Extracted outcomes included AUROC, sensitivity, specificity, cancer type, analyte class, and AI model. Pooled AUROC and absolute AUROC differences between AI-based and non-AI approaches were estimated with 95% confidence intervals using a random-effects model. AUROC values were logit-transformed prior to pooling to stabilize variance and were subsequently back-transformed for presentation. Statistical heterogeneity was quantified using the I² statistic.

**Results:**

Twenty-eight studies across multiple tumor types met inclusion criteria, of which ten provided extractable data for quantitative synthesis. AI-enhanced liquid biopsy achieved a pooled AUROC of 0.924 (95% CI, 0.879–0.953). When head-to-head comparisons were available, AI-based models demonstrated an absolute AUROC improvement of 0.025 (95% CI, 0.019–0.030) compared with conventional analytical approaches. While numerically modest, this improvement was consistent across cancer types and reflects AI’s ability to extract complementary diagnostic signal from complex circulating biomarkers. Between-study heterogeneity was substantial (I² = 88.8%).

**Conclusions:**

AI-enhanced liquid biopsy achieved a pooled AUROC of 0.924 (95% CI, 0.879–0.953), reflecting AI’s ability to integrate multiple weak and complementary biological signals rather than reliance on single circulating biomarkers. These findings support AI’s potential role as a clinical decision- support tool in molecular diagnostics; however, translation into practice will require prospectively validated, clinically calibrated models aligned with specific diagnostic intents.

**Systematic Review Registration:**

https://www.crd.york.ac.uk/prospero/, identifier CRD420251163071.

## Introduction

Early and accurate cancer detection remains one of the central challenges in oncology (1). Conventional tissue biopsy, while the gold standard for diagnosis and molecular profiling, is invasive, non-repeatable, and often fails to capture the spatial and temporal heterogeneity of tumors (2). In contrast, liquid biopsy, the analysis of tumor-derived components in blood or other body fluids—has emerged as a transformative, minimally invasive approach that allows real-time monitoring of tumor evolution (3). Circulating analytes such as cell-free DNA (cfDNA), circulating tumor DNA (ctDNA), cell-free RNA (cfRNA), exosomes, proteins, and circulating tumor cells (CTCs) collectively provide a rich molecular snapshot of the tumor’s genomic and epigenomic landscape (4). Despite its promise, liquid biopsy faces several limitations that hinder clinical translation. The abundance of tumor-derived analytes in peripheral blood is often exceedingly low, especially in early-stage or minimal residual disease settings (5). Moreover, biological noise, pre-analytical variability (e.g., blood-tube type, processing time, freeze-thaw cycles), and assay-dependent biases can substantially affect data quality. These factors reduce the sensitivity and specificity of detection, especially when relying on predefined biomarkers or univariate thresholds. Consequently, while liquid biopsy is increasingly used for mutation tracking and treatment monitoring, its stand-alone diagnostic utility remains limited (6).

To overcome these barriers, the integration of artificial intelligence (AI) and machine learning (ML) into liquid biopsy analysis has attracted growing attention. AI models can leverage complex, high- dimensional datasets and detect nonlinear relationships among genomic, epigenomic, and proteomic features that traditional statistical methods cannot capture. Techniques such as convolutional neural networks (CNNs), gradient-boosting ensembles, and deep multimodal architectures have demonstrated the ability to uncover hidden patterns within cfDNA fragmentation profiles, methylation signatures, and multi-omics fusion data. In particular, deep learning applied to cfDNA fragmentomics has shown potential to distinguish cancer from healthy states even at early stages, achieving diagnostic accuracies previously unattainable with conventional pipelines (7).

Several proof-of-concept studies across NSCLC, colorectal, breast, and hepatobiliary cancers have reported promising AUROC values, often exceeding 0.90, suggesting that AI-enhanced liquid biopsy could meaningfully improve both sensitivity and specificity (8). However, the published evidence remains fragmented. Differences in tumor type, analyte, data preprocessing, AI model architecture, and validation strategy complicate direct comparisons and obscure the true extent of AI’s diagnostic benefit. Moreover, many individual studies are limited by small sample sizes, lack of external validation, and inconsistent reporting of key metrics such as calibration and decision- curve utility (9).

To date, no comprehensive, quantitative synthesis has systematically evaluated the diagnostic performance of AI-based liquid biopsy across cancer types. Such an analysis is needed to clarify the magnitude of improvement over conventional methods, assess reproducibility, and identify methodological sources of heterogeneity (5).

In this systematic meta-analysis, we conducted a rigorous quantitative evaluation of the diagnostic performance of artificial intelligence (AI)-enhanced liquid biopsy models published between 2022 and 2025. Our overarching goal was to provide an integrated and methodologically robust synthesis of the current state of AI-assisted molecular diagnostics.

We first estimated the pooled area under the receiver operating characteristic curve (AUROC) across the studies eligible for quantitative synthesis using a random-effects model, then quantified the mean improvement in diagnostic accuracy attributable to AI integration by comparing AI-based models with conventional, non-AI analytical approaches. Finally, we explored sources of heterogeneity related to tumor type, biomarker class, and validation strategy, and outlined key methodological and translational implications to guide future prospective trials (10–13).

## Methods search strategy and eligibility criteria

We searched studies published between 2022 and 2025 evaluating artificial intelligence, machine learning or deep learning applied to liquid-biopsy data for cancer diagnosis. The literature search was conducted in PubMed, Scopus and Web of Science using a predefined strategy combining terms related to liquid biopsy, AI methods, cancer, and diagnostic accuracy. The core search string used in PubMed was: (“liquid biopsy” OR “circulating tumor DNA” OR ctDNA OR “circulating tumor cells” OR CTC OR “cell-free DNA” OR cfDNA OR exosomes) AND (“artificial intelligence” OR “machine learning” OR “deep learning” OR “neural network*”) AND (cancer OR neoplasm* OR tumor OR oncology) AND (diagnos* OR “diagnostic accuracy” OR sensitivity OR specificity OR AUROC), with filters applied for human studies and publication years 2022–2025.

Equivalent syntax was adapted for Scopus and Web of Science to ensure comprehensive retrieval across databases.

**Figure 1:**
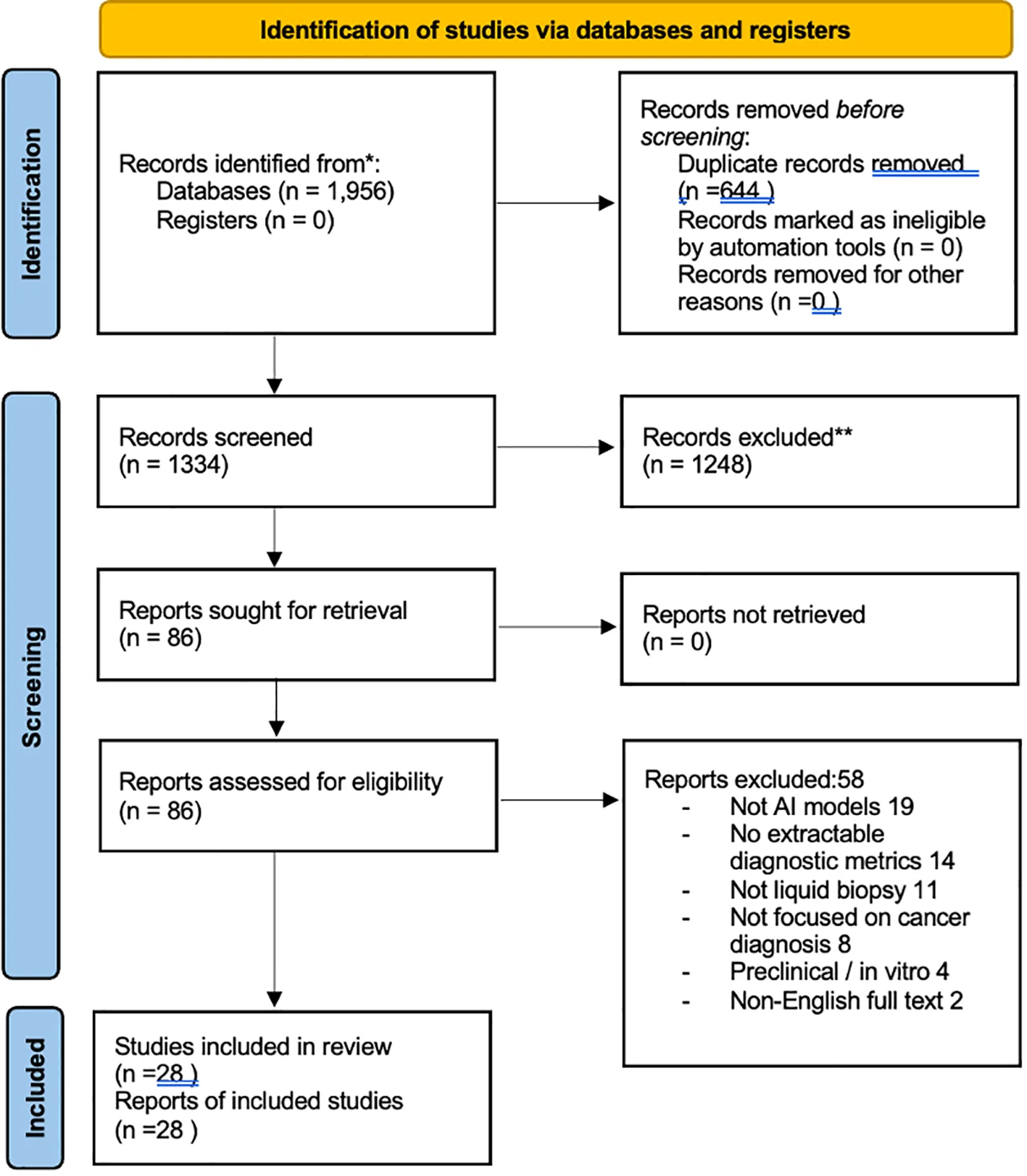
PRISMA 2020 flow diagram illustrating identification, screening, eligibility, and Inclusion of studies.

Eligible studies included human participants, reported at least one diagnostic performance metric such as AUROC, sensitivity or specificity, and used a clearly defined reference standard. We excluded animal or *in-vitro* studies, those lacking extractable diagnostic performance metrics, and studies providing insufficient methodological detail to allow formal appraisal. This review was prospectively registered in PROSPERO (CRD420251163071). Study selection followed PRISMA 2020 guidelines, and the full selection process is presented in the PRISMA flow diagram (**Figure 1**).

Only studies reporting extractable single-point AUROC estimates with sufficient information to derive variance were eligible for quantitative pooling; studies reporting only cross-validation averages or incomplete performance metrics were summarized qualitatively.

### Data extraction and quality assessment

Two reviewers, L.S. and M.G.P, independently and in duplicate extracted study characteristics, including tumor type, analyte class, AI model/architecture, sample size, and validation strategy (cross-validation, train/test split, or external validation). Diagnostic metrics (AUROC, sensitivity, specificity) were collected when available.

Risk of bias was assessed using QUADAS-2, evaluating patient selection, index test, reference standard, and flow/timing domains. Reporting quality for AI models was cross-checked using elements from emerging guidance, including TRIPOD-AI and STARD-AI.

### Statistical analysis

**Figure 2:**
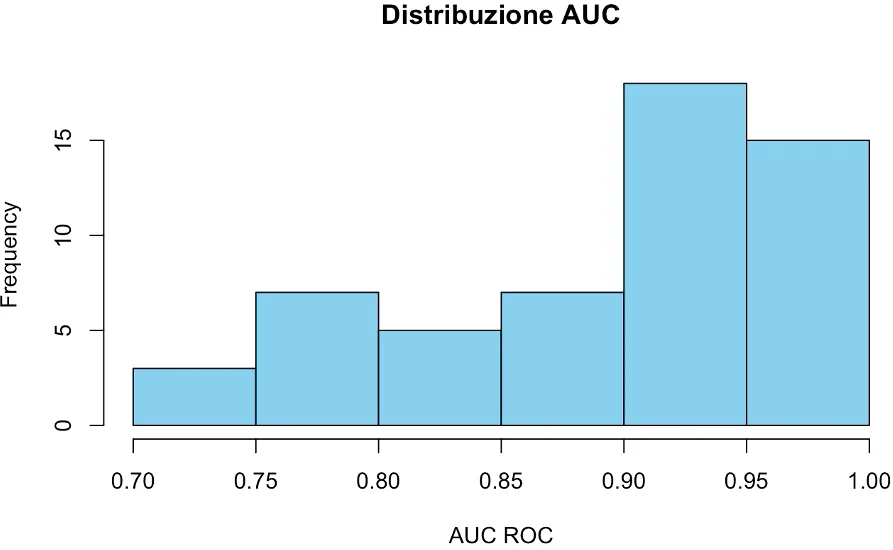
Histogram showing the distribution of AUROC values across the included studies. Most models achieved high diagnostic accuracy, with values clustering above 0.90.

**Figure 3:**
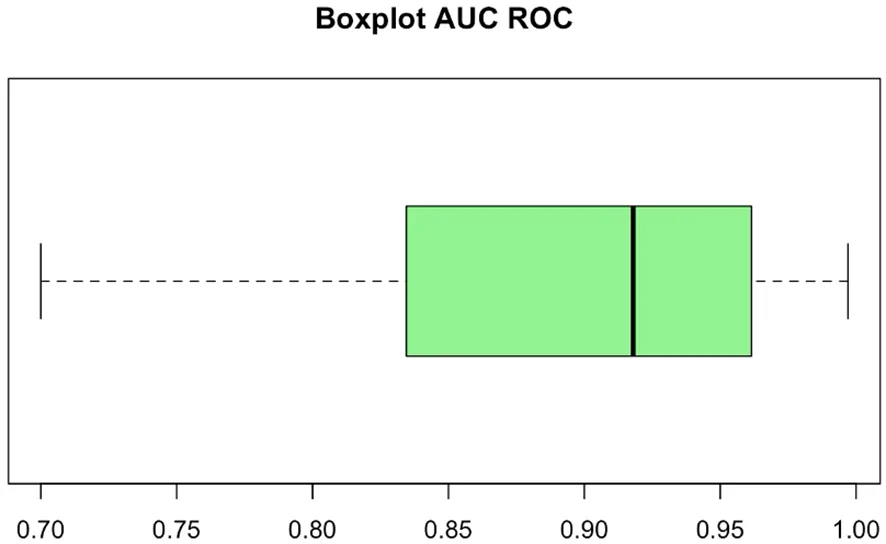
Boxplot summarizing the distribution of AUROC values across included studies, showing a median close to 0.91 and an interquartile range spanning high diagnostic performance.

Pooled diagnostic accuracy was estimated using a Hartung–Knapp random-effects model. Effect sizes included AUROC and the absolute AUROC difference (AI minus non-AI) when a comparator was reported. Heterogeneity was quantified using Cochran’s Q and I² statistics. Descriptive distributions of AUROC values were shown using a histogram and boxplot (**Figures 2**, **3**).

**Figure 4:**
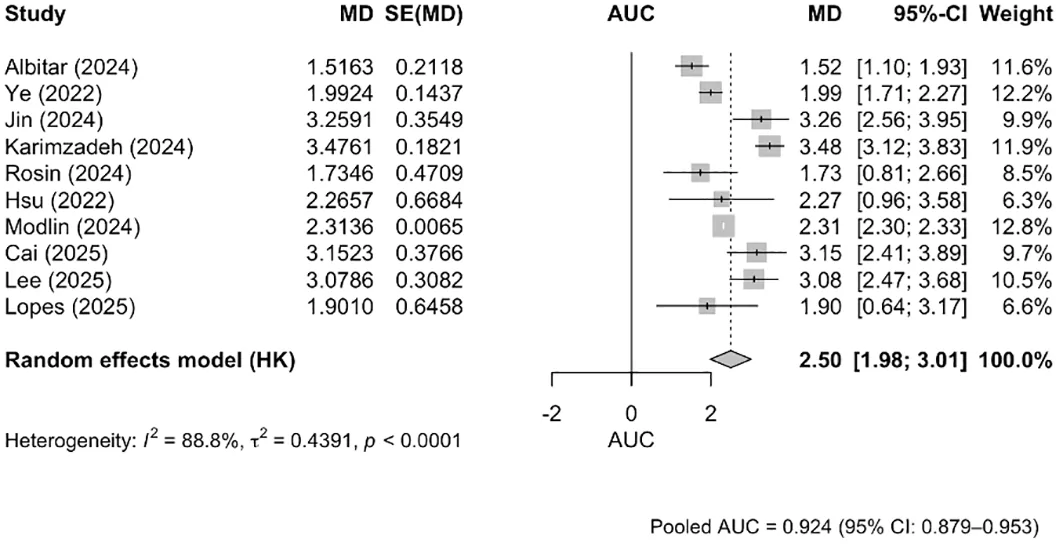
Forest plot showing study-level AUROC estimates with 95% confidence intervals and the pooled random-effects summary (AUROC = 0.924, 95% CI 0.879–0.953).

Descriptive analyses (**Figures 2**, **3**) were conducted across all 28 included studies. The forest plot and pooled AUROC estimate (**Figure 4**) reflect only the 10 studies eligible for quantitative meta- analysis.

A forest plot (**Figure 4**) displays study-level AUROC estimates and the pooled effect. Subgroup analyses were performed, where sufficient data were available, by tumor type, analyte class, and validation rigor. Meta-analysis of calibration metrics, threshold-specific performance, and decision- analytic outcomes was not feasible due to inconsistent reporting across studies.

### Study characteristics

Twenty-eight studies published between 2022 and 2025 were included, encompassing more than 18,000 patients across NSCLC, colorectal, breast, gastric, pancreatic, prostate, hepatobiliary, and multi-cancer settings. Liquid-biopsy analytes included cfDNA mutations, methylation signatures, fragmentomics, ctDNA panels, exosomal RNA, proteomics, and multi-analyte fusion assays.

Most studies used supervised machine-learning models—including random forests, gradient boosting, support-vector machines, and deep neural networks—while others implemented multimodal architectures integrating liquid biopsy with radiomics or clinical variables.

Of the 28 studies, only 10 provided extractable single-point AUROC estimates suitable for quantitative synthesis and were included in the meta-analysis; the remaining studies were summarized qualitatively (**Table 1**). Most excluded studies lacked extractable single-point AUROC estimates or reported only cross-validation averages without sufficient variance information, precluding quantitative pooling. Although these studies contributed important qualitative insights regarding emerging AI methodologies and multi-omic integration strategies, their exclusion from the meta-analysis may have influenced the precision and generalizability of the pooled estimates. The main characteristics of the included studies are summarized in **Table 1**.

**Table 1:** Summary of key characteristics of the studies included in the meta-analysis, including cancer type, study design, AI model, comparator, and diagnostic performance metrics (AUROC, sensitivity, and specificity).

Author (Year)	Cancer Type	N Patients	AI Patients	Study Design	AI Model	Comparator	Outcome	AUROC (AI)	95% CI	Sensitivity (AI)	Specificity (AI)
Albitar (2024) (14)	Multiple solid tumors	1009	641	Retrospective Bayesian ML	Bayesian ML on cfDNA	None	Diagnosis	0.820	0.760–0.879	NA	NA
Ye (2022) (15)	Early-stage NSCLC	1663	728	Prospective multicenter	3D U-Net + ML	Mayo Clinic; VA model	Early lung cancer	0.880	0.852–0.910	89.53%	81.31%
Jin (2024) (16)	Lung cancer screening	328	328	Prospective case-control	SVM + Voxel CNN	LB-only	Nodule risk	0.963	0.946–0.986	–	–
Karimzadeh (2024) (17)	NSCLC	302	302	Prospective	Random forest + cfDNA	None	Diagnosis	0.95	0.946–0.964	NA	NA
Rosin (2024) (18)	Prostate cancer	509	417	Prospective analytic cohort	PROSTATEx AI (ML)	PSA testing	Prostate cancer detection	0.850	0.750–0.950	75%	82%
Hsu (2022) (19)	Hepatocellular carcinoma	297	297	Prospective cohort	Deep learning + radiomics	Radiologist	Tumor response detection	0.906	0.827–0.985	NA	NA
Modlin (2024) (20)	Prostate cancer	795	550	Retro–prospective	Ensemble ML (RF, SVM, XGBoost)	PSA; BPH	Diagnosis / MRD	0.910–0.980	0.913–0.979	83–94%	46–100%
Cai (2025) (21)	Gastric cancer	1595	318	Prospective	AI ctDNA panel ML	CA19-9	Diagnosis	0.957	0.918–0.980	91.2%	95.9%
Lee (2025) (22)	Colorectal cancer	279	279	Real-world registry	ML on biopsy tissue GVH	Biopsy	Stage/diagnosis	0.752	0.663–0.830	NA	NA
Lopes (2025) (23)	Prostate cancer	1751	1397	Case-control	AI cfRNA panel	Healthy vs cancer	Diagnosis	0.86	0.78–0.91	68–99%	99%

### Pooled diagnostic performance

Across the 10 meta-analyzed studies, AI-enhanced liquid biopsy achieved a pooled AUROC of 0.924 (95% CI: 0.879–0.953), indicating high discriminative performance across heterogeneous clinical contexts.

When head-to-head comparators were available, AI models demonstrated an absolute AUROC increase of approximately 0.025, a numerically modest but consistent effect that likely reflects improved integration of complementary biological information rather than a clinically transformative gain per se. Sensitivity reached as high as 99.2% in targeted early-detection cohorts, although specificity varied widely across cancer types and assay platforms. Between-study heterogeneity was substantial (I² = 88.8%).

### Distribution and study-level effects

The distribution of AUROC values was right-skewed, with most models achieving AUROC ≥ 0.90 (**Figure 2**). The median AUROC was approximately 0.91, with an interquartile range of 0.85–0.96 (**Figure 3**).

Study-level AUROC estimates and corresponding 95% confidence intervals are shown in **Figure 4**, along with the pooled random-effects summary estimate.

### Risk of bias

Risk of bias was moderate across the included studies. Using QUADAS-2, the principal concerns involved patient selection, as many studies relied on case–control or enriched cohorts, potentially inflating diagnostic performance. Additional issues were noted in the index test domain, where AI workflows were often reported without full details on preprocessing, threshold selection, or calibration.

The reference standard was generally appropriate (histopathology, radiologist consensus, or clinical follow-up), although variability across tumor types introduced inconsistency. Flow and timing were incompletely reported in several studies, particularly regarding the interval between liquid-biopsy sampling and the diagnostic reference standard.

Overall, applicability concerns were mainly related to heterogeneity in analytes, AI architectures, and clinical settings. Study quality was acceptable but variable, underscoring the need for standardized AI reporting frameworks and prospective validation. Potential risks related to data leakage and model overfitting were difficult to fully assess. Several studies did not provide sufficient detail on preprocessing steps, feature selection, or patient-level separation between training and validation datasets.

### Limitations

This study has several limitations. First, substantial heterogeneity was observed across cancer types, analytes, and AI architectures. More detailed subgroup analyses were constrained by the relatively small number of studies available within individual tumor types and analyte categories, as well as by substantial methodological heterogeneity across AI pipelines and validation approaches.

Second, most included studies were retrospective or used enriched case–control cohorts, which may overestimate diagnostic performance. Third, reporting of AI model development was often incomplete, particularly regarding calibration, threshold selection, and external validation. Fourth, liquid-biopsy platforms varied markedly in pre-analytical processing and assay technology, reducing cross-study comparability.

### Publication bias considerations

Finally, publication bias cannot be excluded, as studies with negative or underperforming models may be less likely to be published. Formal publication bias assessment methods, including funnel plot inspection and Egger regression analysis, were considered. However, only a limited number of studies were eligible for quantitative synthesis, and substantial methodological heterogeneity was observed across tumor types, analyte classes, AI architectures, and validation strategies. Therefore, formal publication bias analyses were considered to have limited interpretability and statistical reliability in the present context.

When head-to-head comparisons were available, AUROC differences were computed at the study level. However, correlation between AI and non-AI models within the same dataset could not be consistently accounted for due to incomplete reporting, and results should therefore be interpreted accordingly.

## Discussion

In this systematic meta-analysis of 28 contemporary studies, artificial intelligence (AI) applied to liquid-biopsy data showed high diagnostic performance across cancer types, with a pooled AUROC of 0.924 (95% CI 0.879–0.953), supporting AI’s potential role not merely as a technical analytical enhancement, but as a clinical decision-support tool in molecular oncology. Where head-to-head comparators were available, AI models outperformed non-AI pipelines, with an absolute AUROC increase of 0.025, indicating a modest but consistent improvement in overall diagnostic discrimination (11, 24).

These findings indicate that AI can extract additional signal from circulating biomarkers beyond conventional analytical approaches, particularly in complex multi-omic settings. From a clinical standpoint, however, these results remain provisional, as diagnostic performance varied substantially across studies and heterogeneity was high (I² = 88.8%), reflecting differences in tumor types, disease stages, analytes, assay technologies, and pre-analytical workflows that influence cfDNA yield and integrity (25).

However, AUROC alone provides only a partial representation of clinical reality, as it summarizes discrimination across thresholds while neglecting prevalence, calibration, and real-world decision boundaries. Accordingly, models with excellent AUROC may still perform suboptimally in low- prevalence populations (26). To support clinically actionable cutoffs, future AI–liquid biopsy studies should routinely report predictive values, calibration metrics, likelihood ratios, and decision- curve analysis (DCA). Translational readiness therefore requires not only algorithmic excellence but also a clear understanding of the clinical consequences of classification errors, both false positives and false negatives (27). In high-stakes settings such as lung-cancer screening or minimal residual disease (MRD) detection, many algorithms prioritize sensitivity over specificity to minimize missed cases, often at the expense of increased false positives. While this trade-off aligns with clinical caution, its downstream clinical and economic consequences should not be underestimated. Clinical deployment should therefore be guided by context-specific threshold calibration to ensure alignment with diagnostic intent and target populations (28, 29). Many studies relied on retrospective case–control designs contrasting advanced cancers with healthy controls, which tend to overestimate diagnostic accuracy and limit applicability to real-world screening or surveillance settings (30). Validation strategies also varied widely, from simple train–test splits to external or prospective cohorts, contributing to spectrum and overfitting bias. Together, these limitations highlight the need for standardized pre-analytics, harmonized assays, and consistent validation practices across laboratories. While AUROC provides a convenient global summary, it is insufficient alone to guide clinical decision-making, as identical AUROC values may correspond to markedly different sensitivity–specificity trade-offs and do not reflect prevalence-dependent metrics such as predictive values (26, 27).

This issue is particularly relevant in low-prevalence contexts such as early detection or minimal residual disease, where even highly discriminative models may generate substantial false-positive rates. Accordingly, threshold-specific sensitivity, specificity, calibration metrics, and decision-curve analysis should be routinely reported to support clinical implementation (28).

From a biological perspective, AI’s advantage is mechanistically plausible: fragmentomics, methylation, mutational burden, nucleosomal architecture, exosomal cargo, and proteomic patterns provide orthogonal and nonlinear signals that deep-learning and ensemble models are well suited to integrate (30, 31). These architectures capture subtle features of tumor–host interactions, helping explain improved discrimination even when univariate biomarkers perform poorly.

Translational progress will therefore require clear definition of intended use, screening, diagnostic triage, or post-treatment monitoring, as each context entails distinct decision thresholds and risk– benefit trade-offs. From a clinical standpoint, AI-enhanced liquid biopsy models should be interpreted according to their intended application, screening, diagnostic triage, or minimal residual disease detection, each of which requires distinct calibration strategies, validation designs, and acceptable trade-offs between sensitivity and specificity.

Real-world adoption also depends on workflow feasibility, turnaround time, cost, and alignment with existing diagnostic pathways. Long-term deployment requires model governance (MLOps), including drift detection, recalibration, and version control (32). Clinician trust remains essential; interpretable outputs and explainability tools can help bridge algorithmic inference and clinical reasoning (33).

Equity and fairness represent additional priorities, as cfDNA quantity and fragmentation patterns vary by ancestry, sex, and comorbidities, and models trained on demographically narrow datasets may underperform in underrepresented populations (34, 35). Privacy-preserving approaches, such as federated learning, may facilitate broader data inclusion without compromising genomic confidentiality (36). Ultimately, maturation of the field will depend on methodological rigor and coordinated prospective evaluation. Multicenter studies using locked algorithms, standardized pre- analytical protocols, predefined clinical endpoints, and transparent reporting frameworks (TRIPOD- AI, STARD-AI) are essential to ensure reproducibility and real-world utility (27, 37, 38). The development of benchmark datasets curated by independent consortia would further enable fair comparison across emerging models (39–41). If prospectively validated, AI-enhanced liquid biopsy may meaningfully improve early detection, risk stratification, and longitudinal monitoring in oncology, supporting a shift toward minimally invasive, data-informed cancer care (42–47).

## Conclusions

This systematic review and meta-analysis shows that liquid biopsy approaches incorporating artificial intelligence achieve high diagnostic discrimination across cancer types, with a pooled AUROC of 0.924 (48, 49). The performance advantage over non-AI analytical pipelines is modest but consistent, and appears driven by improved integration of heterogeneous biological signals rather than intrinsic algorithmic superiority.

However, substantial methodological and biological heterogeneity, together with predominantly retrospective study designs, limit direct clinical translation. Current evidence therefore supports AI as a computational enabler for signal integration rather than a standalone diagnostic solution.

Prospective multicenter validation with locked models, standardized pre-analytical workflows, and adherence to reporting frameworks such as TRIPOD-AI and STARD-AI will be essential before routine clinical implementation (50, 51). Future prospective studies should prioritize clearly defined clinical endpoints, predefined and clinically calibrated decision thresholds, and robust external validation in real-world populations to ensure safe and meaningful integration into routine oncology practice.

## Data Availability

The original contributions presented in the study are included in the article/supplementary material. Further inquiries can be directed to the corresponding author.
