# Supplementary material for: Artificial intelligence–enabled liquid biopsy in cancer: a systematic review and meta- analysis of diagnostic performance and biological implications
Source: Front Oncol. 2026 Jun 17;16:1850705. doi: 10.3389/fonc.2026.1850705 (PMC13318569; doi:10.3389/fonc.2026.1850705)
Supplement: Supplementary file 1 [file Table1.docx]

**Supplementary Table 1**

| Study | Patient Selection | Index Test | Reference Standard | Flow and Timing | Overall |
| --- | --- | --- | --- | --- | --- |
| Albitar (2024) | Unclear | Unclear | Low | Unclear | Unclear |
| Abbosh (2023) | Low | Low | Low | Low | Low |
| Yang (2024) – Gallbladder | Low | Low | Low | Low | Low |
| Ye (2022) | Low | Low | Low | Low | Low |
| Jin (2024) | Unclear | Unclear | Low | Unclear | Unclear |
| Karimzadeh (2024) | Low | Unclear | Low | Low | Unclear |
| Rosin (2024) | Low | Low | Low | Low | Low |
| Hsu (2022) | Low | Unclear | Low | Low | Unclear |
| Modlin (2024) | Unclear | Unclear | Low | Unclear | Unclear |
| Cai (2025) | Low | Low | Low | Low | Low |
| Zeng (2024) | Low | Unclear | Low | Low | Unclear |
| Zomea (2024) | Unclear | Unclear | Low | Unclear | Unclear |
| Thakur (2023) | Low | Low | Low | Low | Low |
| Wang (2024) | Low | Unclear | Low | Low | Unclear |
| Pickering (2024) | Low | Unclear | Low | Unclear | Unclear |
| Ma (2023) | Low | Low | Low | Low | Low |
| Li (2024) | Unclear | Unclear | Low | Unclear | Unclear |
| O’Neill (2023) | Low | Unclear | Low | Low | Unclear |
| Park (2025) | Unclear | Unclear | Low | Unclear | Unclear |
| Kand (2024) | Low | Low | Low | Low | Low |
| Miao (2024) | Low | Low | Low | Low | Low |
| Luo (2025) | Low | Low | Low | Low | Low |
| Varikkolai (2024) | Low | Unclear | Low | Unclear | Unclear |
| Lopek (2025) | High | Unclear | Low | Unclear | High |
| Sekar (2025) | Unclear | Unclear | Low | Unclear | Unclear |
| Yang (2024) – Prostate | Low | Low | Low | Low | Low |
| Kwon (2024) | Low | Unclear | Low | Low | Unclear |

ST1 Risk of bias was assessed across four domains (patient selection, index test, reference standard, and flow and timing) following QUADAS-2 guidance. Overall judgement was considered high if at least one domain was rated as high risk, low if all domains were low risk, and unclear otherwise.
