# Supplementary material for: Artificial intelligence–enabled liquid biopsy in cancer: a systematic review and meta- analysis of diagnostic performance and biological implications
Source: Front Oncol. 2026 Jun 17;16:1850705. doi: 10.3389/fonc.2026.1850705 (PMC13318569; doi:10.3389/fonc.2026.1850705)
Supplement: Supplementary file 2 [file Table2.docx]

**Supplementary Table 2**

| **Study** | **Cancer Type** | **Total Patients** | **AI Cohort** | **Study Design** | **AI Model** | **Comparator** | **Clinical Endpoint** | **AUROC (AI)** | **95% CI** | **AUROC (Comp)** | **Sens (AI)** | **Sens (Comp)** | **Spec (AI)** | **Spec (Comp)** |
| --- | --- | --- | --- | --- | --- | --- | --- | --- | --- | --- | --- | --- | --- | --- |
| Albitar 2024 | Multiple | 1009 | 641 | Retrospective | Bayesian ML | None | Diagnosis | 0.820 | 0.760–0.879 | – | – | – | – | – |
| Abbosh 2023 | Early NSCLC | 197 | 197 | Prospective | ML (CCF) | Standard MRD | MRD detection | HR 5.3 | 2.9–9.7 | – | 94% | 100% | – | – |
| Yang 2024 (Gallbladder) | Gallbladder | 301 | 301 | Prospective multicenter | XGBoost + cfDNA | Benign lesions | Diagnosis | 0.970 | 0.92–1.00 | – | 98.3% | 96.1% | – | – |
| Ye 2022 | Early NSCLC | 1663 | 728 | Prospective multicenter | 3D U-Net + AI | Mayo, VA | Early detection | 0.880 | 0.852–0.910 | 0.895 | 89.5% | 86.3% | 81.3% | 83.3% |
| Jin 2024 | Lung cancer | 328 | 328 | Screening study | SVM + CNN | LB only | Diagnosis | 0.963 | 0.946–0.986 | – | 86.3% | 70.1% | 83.3% | – |
| Karimzadeh 2024 | NSCLC | 302 | 302 | Prospective | RF + cfDNA | None | Diagnosis | 0.950 | 0.946–0.964 | – | 98.3% | 96.3% | – | – |
| Rosin 2024 | Prostate | 509 | 417 | Prospective | PROSTATEx ML | PSA | Diagnosis | 0.850 | 0.750–0.950 | – | 75% | – | 82% | – |
| Hsu 2022 | HCC | 297 | 297 | Prospective | DL + radiomics | Radiologist | Tumor response | 0.906 | 0.827–0.985 | – | – | – | – | – |
| Modlin 2024 | Prostate | 795 | 550 | Mixed design | Ensemble ML | PSA, BPH | Diagnosis + MRD | 0.910–0.980 | 0.913–0.979 | – | 83–94% | – | 46–100% | – |
| Cai 2025 | Gastric | 1595 | 318 | Prospective | AI ctDNA ML | CA19-9 | Diagnosis | 0.957 | 0.918–0.980 | – | 91.2% | – | 95.9% | – |
| Zeng 2024 | Pancreatic | 378 | 318 | Prospective | LASSO + ML | CEA | Early diagnosis | 0.920 | 0.877–0.955 | – | – | – | – | – |
| Zomea 2024 | Multiple | 212 | 205 | Cross-sectional | Discriminant model | None | Diagnosis | 0.884 | – | – | 97% | – | 80% | – |
| Thakur 2023 | NSCLC | 120 | 120 | Prospective | SVM, RF, PCA-LDA | Radiologist | Diagnosis | 0.900 | – | – | 87% | 78% | 85% | 82% |
| Wang 2024 | Thyroid | 147 | 147 | Prospective | Deep ML | Molecular risk | Diagnosis | 0.980 | 0.97–0.99 | – | 92% | – | 86% | – |
| Pickering 2024 | Metastatic GI | 259 | 202 | Prospective | AI-assisted cfDNA | LB | Overall survival | 0.740 | – | – | – | – | – | – |
| Ma 2023 | CRC | 259 | 259 | Prospective | DL cfDNA | TMB | Diagnosis | 0.948 | 0.874–1.00 | – | 85% | 78.7% | – | – |
| Li 2024 | Early NSCLC | 294 | 294 | Retrospective | ML | Radiologist | Diagnosis | 0.948 | – | – | – | – | – | – |
| O’Neill 2023 | Multi-cancer | 524 | 524 | Prospective | V-Deep + ML | Plasma | Diagnosis | 0.870 | – | – | 91% | – | 87% | – |
| Park 2025 | CRC | 238 | 238 | Cross-sectional | LSTM | CEA | CRC detection | 0.955 | 0.932–0.977 | – | – | – | – | – |
| Kand 2024 | Pancreatic | 2568 | 2442 | Prospective | Random Forest | CA19-9 | Diagnosis | 0.955 | 0.900–0.990 | – | – | – | – | – |
| Miao 2024 | NSCLC | 273 | 273 | Multicenter | Deep cfDNA + ML | Radiologist | Diagnosis | 0.910 | 0.89–0.93 | – | 90% | – | 89% | – |
| Luo 2025 | Prostate | 312 | 312 | Prospective | SVM | PSA | Diagnosis | 0.914 | 0.852–0.960 | – | 88% | – | 82% | – |
| Varikkolai 2024 | Gastric | 49 | 29 | Prospective | ML model | CEA | Diagnosis | 0.744 | – | – | – | – | – | – |
| Lopek 2025 | Multiple | 1751 | 1397 | Case-control | Deep cfRNA | Healthy vs cancer | Diagnosis | 0.860 | 0.78–0.91 | – | 68–99% | – | 99% | – |
| Sekar 2025 | Ovarian | 38 | 28 | Prospective | Artificial NN | Healthy vs OC | Diagnosis | 1.000 | – | – | 100% | – | 99% | – |
| Yang 2024 (Prostate) | Prostate | 238 | 238 | Prospective | VGG-based ML | MRI | Diagnosis | 0.812 | – | – | – | – | – | – |
| Kwon 2024 | Breast | 91 | 91 | Prospective | CNN | Radiologist | Diagnosis | 0.912 | – | – | – | – | – | – |

Abbreviations: NSCLC, non-small cell lung cancer; MRD, minimal residual disease; DL, deep learning; RF, random forest; SVM, support vector machine; Sens, sensitivity; Spec, specificity; Comp, comparator.

**ST2.** Characteristics and diagnostic performance of the 28 studies included in the systematic review. Reported data include study design, cancer type, artificial intelligence (AI) model applied to liquid biopsy analytes, comparator (when available), clinical endpoint, and diagnostic performance metrics (AUROC, sensitivity, and specificity). When comparator-based performance was not reported, values are indicated as not available (–).
